# Brown spider venom toxins: what are the functions of astacins, serine proteases, hyaluronidases, allergens, TCTP, serpins and knottins?

**DOI:** 10.1590/1678-9199-JVATITD-2020-0188

**Published:** 2021-07-12

**Authors:** Luiza Helena Gremski, Fernando Hitomi Matsubara, Hanna Câmara da Justa, Zelinda Schemczssen-Graeff, Antonielle Beatriz Baldissera, Pedro Henrique de Caires Schluga, Isabel de Oliveira Leite, Marianna Boia-Ferreira, Ana Carolina Martins Wille, Andrea Senff-Ribeiro, Silvio Sanches Veiga

**Affiliations:** 1 Department of Cell Biology, Federal University of Paraná (UFPR), Curitiba, PR, Brazil.; 2 Department of Molecular Structural Biology and Genetics, State University of Ponta Grossa (UEPG), Ponta Grossa, PR, Brazil.

**Keywords:** Astacins, Serine proteases, Serpins, Knottins, TCTP, Hyaluronidases, Allergens, Spider venom

## Abstract

Accidents caused by the bites of brown spiders (*Loxosceles*) generate a clinical condition that often includes a threatening necrotic skin lesion near the bite site along with a remarkable inflammatory response. Systemic disorders such as hemolysis, thrombocytopenia, and acute renal failure may occur, but are much less frequent than the local damage. It is already known that phospholipases D, highly expressed toxins in *Loxosceles* venom, can induce most of these injuries. However, this spider venom has a great range of toxins that probably act synergistically to enhance toxicity. The other protein classes remain poorly explored due to the difficulty in obtaining sufficient amounts of them for a thorough investigation. They include astacins (metalloproteases), serine proteases, knottins, translationally controlled tumor proteins (TCTP), hyaluronidases, allergens and serpins. It has already been shown that some of them, according to their characteristics, may participate to some extent in the development of loxoscelism. In addition, all of these toxins present potential application in several areas. The present review article summarizes information regarding some functional aspects of the protein classes listed above, discusses the directions that could be taken to materialize a comprehensive investigation on each of these toxins as well as highlights the importance of exploring the full venom repertoire.

## Background

Accidents involving brown spider bites are endemic in South and Southeast regions of Brazil, where they have caused more than 80,000 notifications over the past ten years [1]. Brown spiders have this name due to the characteristic brown color displayed by their bodies. They are cosmopolitan spiders that are found in all continents, but are more adapted to hot or temperate regions with temperatures ranging from 8 to 43°C [2,3]. These spiders belong to the Sicariidae family and the *Loxosceles* genus, whose name alludes to the fact that these animals have their legs curled or bent during the rest period (*Loxosceles* means folded/slanted legs) [4-6]. There are more than 150 species of *Loxosceles* spiders described in the literature [7]. 

They are sedentary animals with nocturnal habits, not aggressive, that organize irregular webs, and prefer to inhabit dark places [2,3,8]. However, few species have clinical significance. In the United States of America, mainly in the South region, and in Central America the spiders *Loxosceles reclusa, L. arizonica and L. deserta* prevail. In South America *Loxosceles intermedia, L. laeta and L. gaucho* are predominant, which are of great medical importance, especially as they present endemic dissemination in some regions of Brazil, Chile and Peru [3,6,8-10]. 

Accidents caused by the bites of brown spiders (*Loxosceles*) are clinically designated as loxoscelism and are characterized by the appearance, in most victims, of skin lesions at the bite site such as necrosis, edema, ecchymosis and erythema, which spread out to neighboring regions (gravitational spread - the hallmark of loxoscelism). Additionally, a massive inflammatory response at the site of the injury and/or its vicinity is reported, with the participation of neutrophils that seem to be responsible for the damage of tissues seen during envenomation [2,3,11,12]. This clinical condition is histologically characterized as an aseptic coagulative necrosis, which consists of a massive destruction of skin structures without direct involvement of infectious agents [13-15]. This picture has a very intriguing mechanism, since the venom does not directly activate the leukocytes involved in the tissue destruction, but instead causes an initial activation of the endothelium, which in turn indirectly activates the leukocytes [16,17]. Signs like itching and skin rash in the vicinity of the bite site are also reported, which suggest an allergenic component in the venom [3,6,8,10,18,19]. 

At the systemic level, less frequent but more severe alterations are reported, which include intravascular hemolysis, thrombocytopenia, and acute renal failure. These signs can evolve and even lead patients to death [6,8,10,20,21]. Even in small amounts, this venom causes severe effects on patients. It is speculated that a few microliters of venom are injected during the bite, which contains between 20 and 200 micrograms of proteins [3,6,22,23]. The venom of *Loxosceles* is produced by two venom-producing glands located in the cephalothorax region of the spiders. These glands display a holocrine secretion mechanism, being the venom produced by epithelial cells organized in a secretory epithelial monolayer, which release a large number of secretory vesicles containing the synthesized toxins towards the apical domain of the cells and then to the gland lumen [3,24]. 

**Figure 1.**
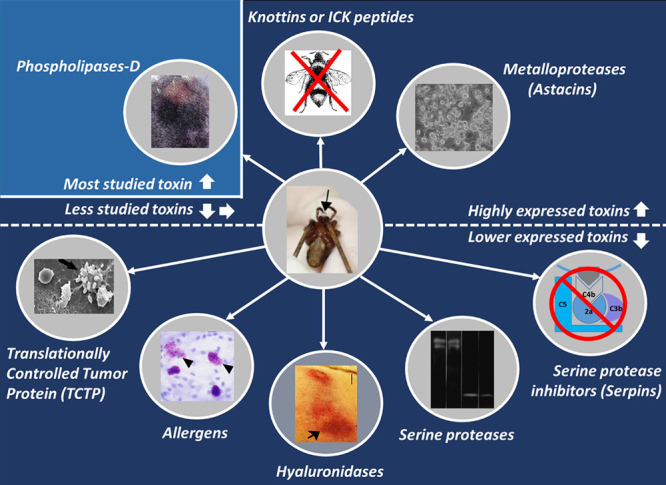
Overview of the toxins present in the venom of *Loxosceles* spiders. Venom components can be divided into two major groups: (i) highly expressed toxins (phospholipases D, knottins or ICK peptides and metalloproteases); and (ii) low-expressed toxins (translationally controlled tumor proteins - TCTP, allergens, hyaluronidases, serine proteases and serine protease inhibitors). **Phospholipases D** can induce all the main effects associated with the whole venom, in addition to displaying insecticidal activity. Recombinant phospholipase D can trigger a dermonecrotic lesion, the hallmark of Loxoscelism (light blue panel - source: Vuitika et al. [123]). In comparison to phospholipases D, all the other venom components have been less studied. **Knottins**, also known as **ICK peptides**, are associated with insecticidal activity. **Metalloproteases** have been linked up to the hydrolysis of extracellular matrix elements facilitating the spread of other toxins, as well as to the induction of deleterious effects on endothelial cells worsening the tissue damage caused by the venom itself (source: da Silveira et al. [40]). **TCTP proteins** act as a histamine-releasing factor, degranulating mast cells and triggering inflammatory events after the envenoming (source: Justa et al. [19]). **Allergens** also participate in the inflammatory process, stimulating the degranulation of mast cells and increasing vascular permeability (source: Justa et al. [19]). **Hyaluronidases** hydrolyze hyaluronic acid and then elicit the gravitational spread of the dermonecrotic lesion (source: Ferrer et al. [68]). **Serine proteases** hydrolyze gelatin and may be involved with the extracorporeal digestion of prey (source: Veiga et al. [51]). **Serpins (serine protease inhibitors)** have been poorly characterized to date, but have already shown to be able to inhibit components (serine proteases) of the complement system. Center image from Chaim et al. [114].

Through omics analyzes (especially proteomics and transcriptomics), it was shown that *Loxosceles* venoms have two groups of toxins: the highly expressed ones, and those expressed in lower amounts (Figure 1). A study regarding the transcripts encoded in the *Loxosceles intermedia* venom-producing glands showed that among the toxins produced in large quantities, knottins (inhibitor cystine knot peptides or ICKs), comprise about 56% of the transcripts that encode toxins, whereas astacins (metalloproteases) represent about 23% of these transcripts, and phospholipases D (dermonecrotic toxins) account for about 20%. Together, the other families of toxins identified to date - hyaluronidases, serine proteases, serpins (serine protease inhibitors), allergens and translationally controlled tumor proteins (TCTP) - account for about 1.3% of the toxin encoding transcripts [25]. 

Among the toxins with high expression, phospholipases D are undoubtedly the most studied and well characterized molecules from the biochemical and functional standpoints. They are highly conserved toxins among the various species of *Loxosceles* spiders described in the literature, comprising a family of toxins with intra- and inter-species occurrence coverage. These toxins represent approximately 16% of the transcripts from the venom-producing glands of *L. laeta* [26], about 15% of the transcripts produced by glands of *L. similis* [27], in addition to the 9% of total transcripts in the glands of *L. intermedia* [25]. They are related to the uncontrolled activation of inflammatory response that appears after envenoming, which results in the skin injury, in addition to triggering the systemic deleterious processes previously mentioned. Herein, phospholipases D will not be explored, since updated data on them were extensively reviewed in recent publications [6,17,28,29]. 

In the present study, we will discuss the literature related to the least studied toxins found in the Loxosceles venoms, which have little data available on their participation in the biology of venoms. Two families of toxins belonging to the group of highly expressed toxins (knottins and astacins) will be addressed as well as the other families of toxins belonging to the group of low-expressed toxins (hyaluronidases, allergens, TCTP, serine proteases, and serpins) (Figure 1). Although these toxins are not individually involved in all venom deleterious activities like phospholipases D, recent studies point out that some of these molecules have a relevant participation in the framework of loxoscelism pathophysiology, in addition to being a potential target for the development of biotechnological resources or for the understanding of molecular and cellular processes. 

## Methodology

This systematic review was elaborated based on articles retrieved from the electronic databases PubMed and Google Scholar. Original articles, review studies and case reports published until January 15, 2021 were considered after being identified using as keywords “*Loxosceles*” and the class of a specific toxin (i.e., metalloprotease, serine protease, hyaluronidase, allergen, TCTP, serpin or ICK peptide) combined. After the identification of the articles, exclusion criteria were sequentially applied in order to select appropriate articles regarding the aim of this review. In the first step, articles repeated between the databases accessed were removed as well as articles with titles showing no relation to the toxins investigated. Only articles in English and Spanish were considered for further analyses whereas theses and dissertations shown by the search engine Google Scholar were also disregarded. 

**Figure 2.**
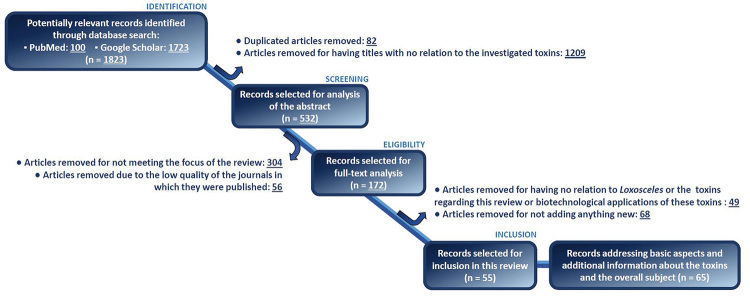
Flow chart highlighting the methodology used in order to produce this systematic review.

Subsequently, the articles selected in the previous step were screened based on their abstract, resulting in the removal of articles that do not contain information supporting the aim of this review or were published in low-quality journals (with no impact factor assigned). The remaining articles were submitted to full-text appreciation followed by discarding of articles that do not involve the context intended (i.e., characterization of specific toxins present in the venom of Loxosceles spiders) or that do not add any new information to the literature of Loxosceles toxins herein described. Finally, the remaining 55 articles were included in this review. Other 65 articles were biased, selected and included since they were fundamental to set the background and historically, structurally, biochemically or biologically characterize each class of toxin. Figure 2 summarizes the steps adopted in order to produce this review.

### Astacins and Serine Proteases

**Figure 3.**
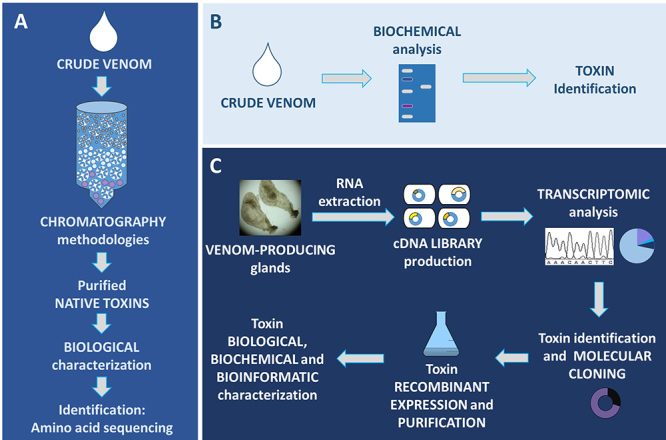
Experimental strategies used to study some toxins present in the venom of *Loxosceles* spiders. **(A)** De Castro et al. [110] reported the obtainment of native toxins (knottins or ICK peptides) purified from *L. intermedia* crude venom by means of sequential chromatographic approaches. The native toxins were then biologically characterized as insecticidal molecules and their amino acid sequences were identified by sequencing. **(B)** Hyaluronidases from *L. intermedia* were first studied by Da Silveira et al. [^66^] using biochemical methods (zymography). These analyses showed molecules displaying hydrolytic activity in the venom upon hyaluronic acid and chondroitin sulfate. Toxin identification using biochemical assays was also described for metalloproteases. Feitosa et al. [^32^] showed that the crude venom of *L. intermedia* degraded human fibronectin in proteolytic digestion kinetic experiments. Further analyses showed that metalloprotease inhibitors blocked crude venom ability to hydrolyze fibronectin, which together with zymography experiments using gelatin as a substrate, pointed out the presence of metalloproteases in the studied venom. **(C)** Identification and characterization of toxins can also be performed using transcriptome analysis, followed by recombinant protein-production techniques and studies for the characterization. Sade et al. [87] and Boia-Ferreira et al. [88] reported biological and biochemical characterization of a recombinant *L. intermedia* TCTP expressed in bacterial model identified in the venom-producing gland transcriptome. Ferrer et al. [^68^] produced a recombinant hyaluronidase in bacterial model followed by *in vitro* and *in vivo* analysis; the sequence coding this hyaluronidase was also identified in the transcriptome of *L. intermedia* venomous gland. In addition, transcriptomic findings revealed a sequence of an allergen encoded in *L. intermedia* venom-producing gland. Based on this information, Justa et al. [19] recombinantly expressed this toxin using baculovirus-infected insect cells and characterized its functionality. Venom producing glands (C) from Chaim et al. [114].

The existence of proteases in brown spider venoms was originally reported over 40 years ago. The first descriptions showing the presence of proteases in the brown spider venoms were made using the crude venom of Loxosceles reclusa, which showed proteolytic activity on larvae of Heliothis viresceus and Musca domestica [30]. These data on proteolytic activities in brown spider venoms were confirmed by the use of synthetic substrates derived from the L-aminoacyl-beta-naphthylamide, which were hydrolyzed by L. reclusa venom [31]. The discovery of metalloproteases (astacins) in the venoms of brown spiders has the participation of several Brazilian researchers, and was described almost twenty years later, in studies that initially used the crude venom from Loxosceles intermedia. Through kinetics experiments of protein substrate degradation using fibronectin or fibrinogen incubated with crude venom, results showed the presence of proteolytic activity on these proteins (Figure 3). 

Zymograms with copolymerized gelatin identified proteases with molecular masses between 30 and 35 kDa, and zymograms with copolymerized fibronectin or fibrinogen showed proteases with 22-28 kDa. In both cases, only divalent metal chelators such as ethylenediaminetetraacetic acid (EDTA) and 1,10-phenanthroline - but not inhibitors of other classes of proteases such as phenylmethylsulfonyl fluoride (PMSF), aprotinin, leupeptin, and pepstatin A - blocked these proteolytic activities, confirming that the proteases involved on such as activities are metalloproteases [32]. The presence of metalloproteases in the venoms of brown spiders were also demonstrated by other authors, who showed metalloprotease-dependent proteolytic activities in *L. intermedia* venom on the substrates entactin and on heparan-sulfate proteoglycan protein core synthesized by endothelial cells [33,34], in addition to proteolytic activity on fibrinogen [35]. In addition, it was found that a metalloprotease of *L. gaucho* venom acts upon red cell band 3 transmembrane protein [36]. Then, two important descriptions were made: the first showing the biological conservation of these metalloproteases in venoms of different species of *Loxosceles* spiders, such as *L. rufescens* [37], *L. gaucho, L. deserta* and *L. laeta* [38], indicating the importance of metalloproteases in the biology of these animals. Another important description used extract of *L. intermedia* venom-producing glands, discarding criticisms of a possible contamination of venoms by the digestive secretions of spiders used in the previous articles, which were obtained by electrostimulation, and then proving that metalloproteases are components of venoms and not digestive contaminants [39]. 

The classification of these metalloproteases as astacins was first described by the cloning of an isoform of these enzymes from a cDNA library obtained from the venom-producing gland of *L. intermedia*, which identified a metalloprotease with 30 kDa, containing a sequence signature of astacins. This metalloprotease - which was named LALP (from *Loxosceles* astacin-like protease) - was obtained in its recombinant form by heterologous expression using bacterial model and proved to have gelatinolytic, fibronectinolytic and fibrinogenolytic properties [40]. Transcriptomic studies of the venom-producing glands of *L. laeta*, which showed the presence of 8.2% of transcripts for metalloproteases [26], and *L. intermedia*, which showed about 9.8% of the transcripts for the same family of toxins, bring into view further evidence that strongly suggests the presence of metalloproteases in different brown spider venoms [25]. Metalloproteases are also expressed in high amounts in the venom gland of *L. gaucho*, as found by a transcriptome analysis [41]. A recent study analyzed the Peruvian *L. laeta* transcripts focusing on LALPs, and found 9 putative sequences coding for astacine-like metalloproteases highly similar to LALP1 from *L. intermedia* [42]. In addition, the authors compared the activities of Brazilian and Peruvian *L. laeta* venom upon fibrinogen and gelatin/collagen, and concluded that the Peruvian venom have a higher activity upon these molecules than the Brazilian one [42]. A proteomic analysis of the *L. intermedia* crude venom using mass spectrometry also described these enzymes as components of the venom [43]. 

Finally, astacins were described as forming an intra and inter-species family of toxins in the brown spider venoms, which contain at least six different isoforms as showed through an elegant experiment using two-dimensional zymogram copolymerized with gelatin [44,45]. These data were later confirmed by molecular cloning of members of metalloprotease family, strengthening the existence of various isoforms of these proteases in the brown spider venoms [46]. The existence of families of astacins in the venoms of brown spiders was also demonstrated by the use of a monoclonal antibody produced against a *L. intermedia* LALP isoform, which cross-reacted with crude venoms of *L. laeta* and *L. gaucho* and neutralized the proteolytic effects of these enzymes in the venoms [47]. The biological conservation and the presence of astacin isoforms in different brown spider venoms have already pointed out the participation of these proteolytic enzymes in the biological events related to brown spider venom. However, what are the true biological functions of these molecules? 

The first aspect that can be discussed is undoubtedly the participation of astacins as regulating molecules of extracorporeal digestion performed on prey captured by these spiders, which are carnivorous and considered very efficient predators that ingest food in liquid form [48]. The presence of these endo-proteases in the venom helps to degrade protein components of the obtained preys, and thus, facilitate the feeding of these spiders. Another possibility is the proteolytic action of these metalloproteases on the components of blood vessel endothelial cells, such as heparan-sulfate proteoglycan, involved in endothelial cell biology and blood vessel stability. This mechanism was previously investigated [34,35] and could induce signs related to blood vessel disorders after envenoming, such as increased capillary permeability, edema, and ecchymosis. Moreover, the action of astacins on extracellular matrix components involved in blood vessel stability, blood clotting, platelet adhesion and aggregation - such as fibronectin, fibrinogen and entactin - could explain other events that occur after envenoming, such as hemorrhage and even the reported difficulty in healing the skin lesions triggered by accidents [32-35]. 

The proteolytic action of brown spider venoms has also been demonstrated on basement membranes, structures with enormous importance in the organization of various tissues [49]. Together with the proteolytic action on soluble components of the extracellular matrix, these activities could explain some toxic effects caused by the venoms as previously discussed. By making the extracellular matrix loosened and disorganized, astacins could also promote the spread of other toxins to the nearby blood vessels and other parts of the bodies of injured victims, enhancing deleterious systemic effects during envenoming. Finally, metalloproteases present in the venoms could act as molecular scissors that cleave precursor molecules of the venom and/ or confined to the hosts, thus activating the toxic activities seen after accidents. An example that illustrate this mechanism is that of zymogens, produced by leukocytes, which could be activated during the unregulated inflammation that occurs after accidents, as previously hypothesized [40]. 

Another interesting work that deserves to be cited and is based on the participation of astacins in the toxicity of brown spider venoms used linear sequences of an astacin from the venom of *L. intermedia* LALP-1 (SLGRGCTDFGTILHE, ENNTRTIGPFDYDSIMLYGAY, and KLYKCPPVNPYPGGIRPYVNV) in the construction of a chimeric recombinant antigen containing, in addition to LALP sequences, the sequences of a hyaluronidase and a phospholipase D present in this venom. This chimera was antigenic and triggered the production of antibodies that neutralized the dermonecrotic activity in rabbits’ skin and inhibited mice lethality induced by the crude venom [50]. Another hybrid immunogen consisting of hydrophilic regions from the metalloprotease LgALP1 from *L. gaucho* and from a phospholipase D of the same species was constructed, expressed and used to produce antibodies in mice. This antiserum neutralized dermonecrotic (*in vivo*) as well as fibrinogenolytic and platelet aggregation (*in vitro*) activities elicited by *Loxosceles* venoms [41]. Astacins, as well as other toxins present in the venom of *Loxosceles* spiders, are not feasible to be obtained in their native form through purification from crude venom because of the low yield of the venom extractions. Moreover, these enzymes are difficult to be recombinantly obtained in their soluble and active form [40,46]. Due to this difficulty in isolating these molecules, the knowledge regarding their participation in the envenoming process is based on theoretical possibilities and/or hypotheses related to their biochemical properties, pointing out the lack of robust evidence for final conclusions.

Other proteases described in brown spider venoms belong to the family of the serine proteases. In this case, literature data are even scarcer and are restricted to a few published articles. The first description of serine proteases as part of brown spider venoms was made using crude venom of *L. intermedia*, which after treatment with thrombin, activated two molecules with gelatinolytic activity at regions of 85 kDa and 95 kDa in a zymogram analysis. These proteases were inhibited by serine proteases inhibitors as PMSF, aprotinin, benzamidine, soybean-trypsin inhibitor and leupeptin, but not by other protease inhibitors as EDTA, 1-10’-phenanthroline, iodoacetamide and pepstatine-A. In addition, such data indicated that these toxins are present in the forms of inactive zymogens, and that after treatment with thrombin, they acquire proteolytic activity [51]. They are enzymes with an optimal pH between 7 and 8 and, interestingly, they do not have a wide spectrum of proteolysis. These enzymes showed proteolytic activity restricted to gelatin, with no catalytic activity on proteins such as BSA, hemoglobin, IgG, casein or laminin [51]. The presence of serine proteases in brown spider venoms was reinforced by transcriptomic analysis of *L. laeta* venom-producing gland, which indicated the presence of serine proteases transcripts comprising about 0.5% [26], and also of *L. intermedia* venomous gland, which revealed about 0.3% of transcripts encoding serine proteases [25]. These data indicate inter-species conservation, strengthening the biological importance of these proteases, although in both cases they are low expressed in comparison to other toxins such as the phospholipases D and ICKs. 

The results based on transcriptomic analysis were confirmed by proteomic studies, which showed the presence of serine proteases in the crude venom of *L. intermedia*, indicating that these molecules are indeed components of brown spider venoms [43]. As reported for astacins, the purification of native serine proteases from *Loxosceles* spiders’ crude venoms is virtually unviable and the production of these enzymes in heterologous models has proved to be quite difficult. For these reasons, the biological functions of serine proteases in the venoms of brown spiders have not been determined yet, leading to only speculative hypothesis regarding their functionality. Based on these theoretical assumptions, as described for the astacins, the serine proteases could act as molecular scissors that work by activating precursors molecules in the crude venoms or in victim’s body, and/or digestive enzymes that participate in the extracorporeal digestion occurring shortly after the envenoming of the prey. However, the precise functions of serine proteases in the envenoming are still to be determined.

### Hyaluronidases

Hyaluronidases are found in several animal venoms including those of spiders [52-56] snakes [57], caterpillars [58], and bees [59,60]. Hyaluronidase activity in brown spider venom was first reported in the venom of *L. reclusa* [61]. Later, other description regarding hyaluronidase in a brown spider venom was made by Wright et al. [62]. In this study, a hyaluronidase was purified from the venom glands of *L. reclusa* and exhibited activity on hyaluronic acid (optimum pH 5.0 - 6.6) and chondroitin sulfate, being the former the preferred substrate. The authors also tested the *in vivo* activity of this enzyme and observed the development of a mild erythema in guinea pigs after 6 hours that subsided over the next 24 hours. Although a complete neutralization of purified *L. reclusa* hyaluronidase by the gamma globulin fraction of a specific antivenom raised against *L. reclusa* crude venom was observed *in vitro*, the whole antiserum exhibited only a slight inhibitory effect on the spreading action of the venom [62]. The presence of antibodies in the antivenom that recognize *L. reclusa* purified hyaluronidase was confirmed later by immunodiffusion studies [63]. 

At that time, toxinologists already understood that hyaluronidases in spider venoms were not a toxic element per se, but probably act as a spreading factor [64,65]. Later, hyaluronidase activity was described in the venom of various Loxosceles species, e.g. L. deserta, L. gaucho, L. intermedia and L. laeta, and this activity appeared in a hyaluronic acid zymogram as a 44 kDa enzyme [38]. L. intermedia hyaluronidase was further characterized by da Silveira et al. (Figure 3B) [66]. These authors showed that this hyaluronidase is a hydrolase characterized as an endo-b-N-acetyl-D-hexosaminidase, has an optimal activity at 6.0-8.0 pH and hydrolyze both hyaluronic acid and chondroitin sulfate in vitro and hyaluronic acid in vivo [66]. Proteomic and transcriptomic analyses identified few sequences that correspond to hyaluronidase, evidencing that they are low-abundance toxins in Loxosceles venom, comprising 0.13% of L. laeta and 0.05% of L. intermedia venom gland transcripts [25,26,67]. 

The role of Loxosceles venom hyaluronidases as spreading factors was first demonstrated when a recombinant L. intermedia hyaluronidase was produced [68]. Dietrich’s hyaluronidase, as it was named, was expressed in E. coli cells and subjected to in vitro refolding in order to obtain a soluble and active 6xHis tagged enzyme with ~45 kDa (Figure 3C). This recombinant enzyme retained linear antigenic determinants from native hyaluronidases of Loxosceles crude venom as demonstrated by immunoassays. Finally, Dietrich’s hyaluronidase increased the area of dermonecrosis and enhanced edema induced by a recombinant phospholipase D, as well as triggered the gravitational spreading of the lesion. These data proved the role of Loxosceles hyaluronidases as a spreading factor of other toxins near the bite site [68]. Two other spider hyaluronidases were produced as recombinant toxins: CsHyal (from Cuppienius salei), which was produced in E. coli and further refolded, and BvHyal (from Brachypelma vagans), which was expressed using baculovirus system in insect cells [69,70]. CsHyal potentiated the insecticidal activity of neurotoxins in invertebrate preys, and authors speculated that this glycosidase may act as a spreading factor that enhance the activity of neurotoxic venom compounds [70]. 

Recently, a novel isoform of *Loxosceles intermedia* venom hyaluronidase was produced in a baculovirus-infected insect cells system and named LiHyal2 [71]. This recombinant glycosidase was produced as an active glycosylated enzyme and the biological characterization of LiHyal2 confirmed its ability in acting as a spreading factor.

By using two linear epitopes of *Dietrich*’s hyaluronidase (NGGIPQLGDLKAHLEKSAVDI and ILDKSATGLRIIDWEAWR) combined with epitopes of other *Loxosceles* toxins (e.g. astacin-like protease and phospholipase D), Lima et al. [50] produced a recombinant multiepitopic protein named rMEPLox (recombinant MultiEpitopic Protein derived from Loxoscelic toxins). Antibodies against this protein efficiently neutralized hyaluronidase activity of *L. intermedia* venom [50]. These results represent one of the various possibilities of using *Loxosceles* hyaluronidases as biotools for therapeutical applications. These enzymes are related to several physiological and pathological processes. In fact, venom hyaluronidases are of interest also because they belong to the same class of mammal hyaluronidases, as mentioned earlier. The use of a recombinant human PH20 hyaluronidase (rHuPH20; Halozyme Therapeutics, Inc.) to overcome the resistance to bulk fluid flow in the subcutaneous space and favor drug delivery, dispersion, and absorption, is currently FDA-approved. It acts by degrading hyaluronic acid, facilitating the route of administration and optimizing the dosage of subcutaneous therapies [72]. 

A recombinant human hyaluronidase was also proved to be effective and secure as a facilitating agent for subcutaneous immunoglobulin in a retrospective, multicenter study (fSCIG; HyQviaR ®) in elderly patients with primary or secondary immunodeficiencies [73]. They are also widely applied in the field of dermatology, to degrade hyaluronic acid filler to reverse cutaneous augmentation with this glycan [74]. In short, the production of recombinant spider venom hyaluronidases is a promising alternative since they can contribute to understand the role of these glycosidases in the venom and to the development of specific therapies to treat loxoscelism, besides having various potential applications for the pharmaceutical industry [75,76].

### Allergens

Accidents involving bites caused by bees and ants, in addition to exposures to animals such as cockroaches and mites can generate serious allergic reactions such as fever, edema, vertigo and anaphylactic shock [77-81]. In spiders, little is known about allergenic molecules and their biological activities. However, in accidents involving spiders from the *Loxosceles* genus, symptoms at the cutaneous tissues as itch, erythema, edema, cutaneous rash, and in some cases acute generalized exanthematous pustulosis are common, suggesting allergic reactions in some instance [6,28,82,83]. The presence of toxins with hyaluronidase activity in the venoms of *Loxosceles* spiders, which are strong allergenic factors in bee venoms, was initially described by studying the crude venom of *L. reclusa* [62]. Later, toxins characterized as hyaluronidases were identified in venoms of other *Loxosceles* species, as mentioned earlier [37,38,66], however there was never a direct correlation of these toxins with allergenic activities in these venoms. 

The first scientific report demonstrating the presence of allergens in venoms of brown spiders was obtained through proteomic analysis of the crude venom of *L. intermedia* using MS/MS mass spectrometry [43]. The authors pointed out the presence of a toxin similar to the mite allergen of group 7 and suggested this toxin could be directly related to allergenic activities seen in injured patients. In this same way, by analyzing the transcriptome of the venom-producing glands of *L. laeta*, it was shown that 0.6% of toxin coding transcripts were related to a toxin characterized as a venom allergen III, which is conserved in animals with an allergenic potential such as the ant *Solenopsis invict*a, the wasps *Vespula vulgaris* and *Dolichovespula maculata* and the mosquito *Aedes aegypti* [26]. This toxin could hypothetically be related, according to the authors, to allergic reactions described during the envenoming caused *L. laeta* venom. 

The presence of allergenic factors in the venoms of *Loxosceles* spiders was also demonstrated in the study regarding the transcriptome of *L. intermedia* venom glands, in which 0.2 % of transcripts encoding toxins were described as being allergens [25]. The similarity of these allergenic toxins from *L. intermedia* with other allergens present in the venoms of the spider *Lycosa sigoriensis* and the scorpoion *Opisthacanthus cayaporum* as well as mite allergens of *Ixodes scapularis* and *Argas monolakensis* suggests the involvement of this toxin in the possible allergic responses seen after accidents. Finally, the existence of an allergen in the venoms of *Loxosceles* spiders was greatly suggested by a study using molecular biology techniques, which reported cloning of an allergen named as LALLT (*Loxosceles* allergen-like toxin) [19]. This toxin was cloned, expressed in the baculovirus/insect cells system, purified and some of its biological activities were reported (Figure 3C). The allergen from *L. intermedia* venom has a molecular mass of 42 kDa and presents epitopes that cross reacted with anti-venom sera developed using crude venoms from *L. laeta* and *L. gaucho*, suggesting that these molecules are conserved in different species of the *Loxosceles* genus. This biological conservation was also revealed by analysis involving multiple sequences alignment of cDNA-deduced amino acid sequences for LALT orthologues from *L. laeta* and from *L. gaucho* venom, reinforcing the existence of a family of allergens in the venoms of brown spiders [19]. 

LALLT has 18 cysteine residues and belong to the CAP superfamily, showing significant identity to other allergens from spiders, scorpions, mites and ticks. Experiments of biological characterization of LALLT showed that this recombinant molecule caused edema in the skin of rabbits, increased vascular permeability and triggered paw edema in mice, besides increasing calcium influx and inducing release of beta-hexosaminidase from mast cells (RBL-2H3) *in vitro*. Finally, LALLT caused degranulation of rat mesentery mast cells [19]. In this same study, histological analysis of the skin of rabbits exposed to recombinant LALLT revealed edema and an infiltrate of inflammatory cells on the dermis, findings that are common in the histopathological analysis of samples from patients with allergies caused by arthropods bites [84]. 

All the data exposed here support the existence of allergens in the venom of *Loxosceles* spiders, providing valuable information that can assist in the treatment of accidents in the hospital or outpatient settings. The fact that these toxins belong to the group of low expressed toxins in *Loxosceles* venoms has to be taken into account, since their low concentrations can make these allergenic responses uncommon, restricted to more susceptible patients. 

### Translationally Controlled Tumor Protein (TCTP)

*Loxosceles intermedia* TCTP protein, LiRecTCTP, was identified in the cDNA library of the brown spider venom gland of *L. intermedia* [25]. This protein exhibits a high degree of similarity with tick TCTPs (~70%), which are described as histamine release factors [85-87]. The complete sequence identified in the cDNA library contains 536 bp, which encodes a 172-amino acid protein with a predicted molecular mass of 22.3 kDa and a pI of 4.7 (mature TCTP) [87]. Additionally, the transcriptome study of the *L. intermedia* venom gland identified 0.2% of the total transcripts corresponding to TCTP protein transcripts [25]. In 2012, Sade et al. [87] performed cloning, heterologous expression, purification and functional characterization of the *L. intermedia* TCTP (Figure 3C). The recombinant protein, expressed in *E. coli* with a 6 His-tag at the N-terminus, was called LiRecTCTP. Purification involved two chromatography steps - an affinity chromatography using Ni-NTA agarose resin with subsequent ion exchange chromatography (DEAE-sepharose) [87]. Recently Boia-Ferreira et al. [88] have standardized a new purification protocol with higher yield and purity using the Akta purified system and affinity chromatography (Ni-NTA agarose). Secondary structures and solubility analyses of LiRecTCTP were performed by using circular dichroism spectroscopy and showed the proper folding features of the recombinant protein [88].

Envenomation by *Loxosceles* spiders can cause hypersensitivity and allergic reactions. The cutaneous symptoms generated by the venom include erythema, edema, itching and pain. Rattmann et al. [89] demonstrated that the *L. intermedia* venom triggers mast cell activation and histamine-dependent effects. Initial inflammation events, such as increased vascular permeability, were related to the participation of histaminergic and serotonergic receptors. As mentioned earlier, many symptoms observed during loxoscelism can be mimicked by phospholipase D toxins (PLDs), the most characterized and studied family of toxins in *Loxosceles* venoms. However, recombinant PLDs are not able to induce paw edema with the same intensity as the crude venom, pointing to histaminergic events in the increased formation of edema during envenomation [6,87]. 

Brown spider venom was shown to be capable of causing regulated release of mast cell mediators, mainly histamine, responsible for inducing vasodilation in experiments with rat aorta using a chamber for an isolated organ [89]. Paludo et al. [90] identified the presence of histamine in the venom in sufficient quantities to exert inflammatory effects. Despite this, the dialyzed venom, without the presence of histamine, was still capable of exerting a certain histamine-dependent inflammatory effect, due to some other component present in the venom, acting directly on mast cells [89,90]. First Sade et al. [87] and then Boia-Ferreira et al. [88] demonstrated that the TCTP of *L. intermedia* participates in the exacerbated inflammatory process resulting from accidents: LiRecTCTP causes *in vivo* increased vascular permeability and edema in mice, in a time and concentration dependent manner [87,88]. Therefore, these results suggest the LiTCTP may be the first and fastest component to induce edema formation in loxoscelism pathophysiology [87]. Boia-Ferreira et al. [88] also demonstrated that LiRecTCTP is capable of activation mast cells (de-sensitized RBL-2H3 cells) leading to degranulation *in vitro*. 

Expression profile analysis via quantitative real-time PCR showed that LiRecTCTP induced the cellular expression of cytokines involved in allergic and parasitic processes such as IL-3, IL-4, and IL-13 in cultured RBL-2H3 cells. *In vivo* assays showed that when LiRecTCTP was injected in mice together with inhibitors of histamine receptors (H1, H2, H3 and H4) a reduction in vascular permeability and edema was observed when compared to isolated toxin, confirming that this toxin is responsible for inducing these deleterious histaminergic effects [88]. H1 and H2 receptors inhibitors (prometazine and thioperamide, respectively) have been shown to significantly reduce the effects of LiRecTCTP on increasing vascular permeability. The degranulation inhibitor cromolyn, in turn, was able to abrogate the edematogenic effect prompted by LiRecTCTP. Furthermore, experiments of dermonecrosis using rabbits demonstrated a synergism between LiRecTCTP and a recombinant phospholipase D toxin. These data emphasizes LiRecTCTP relevant participation in the inflammatory and histaminergic cutaneous effects of loxoscelism for acting as a histamine release factor and thus contributing to the systemic dispersion of other venom components [88]. 

TCTP-related proteins were also identified in the venom of other *Loxosceles* spiders (*L. laeta* and *L. gaucho*) by immunoblot cross-reactivity assays [91]. Literature on TCTP from spiders is scarce but some sequences were identified in the venom of spiders from different species [92-95], and in gland secretions of ixodid tick parasites, which are also arachnids [96]. Concerning the biological and evolutionary purpose of this toxin to be present in *Loxosceles* venom we must highlight that *L. intermedia* TCTP does not present a signal peptide for endoplasmic reticulum translocation, this toxin can be secreted by exosomes and also by the holocrine secretion pathway [24], as other constituents of whole venom [87].

Studies on TCTP as a venom toxin are very few and its biological and evolutionary role as a venom component in prey capture remains still unknown [97]. In contrast, as a multifunctional protein involved in several biological processes, its biotechnological potential is enormous and yet to be further explored.

### Serine Protease Inhibitors (Serpins)

Another group of toxins found in the venoms of *Loxosceles* spiders includes protease inhibitors of the serine protease family characterized as serpins [28,98]. The first evidence in the literature pointing out the existence of protease inhibitors in *Loxosceles* spiders was described using crude venom of *L. reclusa*, which showed the presence of a potent inhibitory activity on the complement-dependent hemolysis (an event highly dependent of serine proteases). This component of the venom showed properties such as not being dialyzable, but can be excluded from the venom by means of a gel filtration chromatography using Sephadex G-75, in addition to being stable under a broad range of pH [99]. Serine protease inhibitors were initially described in the venoms of brown spiders by means of transcriptome analyzes of *L. laeta* venom-producing glands. In this study, transcripts encoding serine protease inhibitors corresponded to 0.6% of the total transcriptome [26]. Serine proteases inhibitors were additionally identified through proteomic analyzes using crude venom of *L. intermedia* that was submitted to sequential chromatography steps by using cation exchange and reverse phase to purify proteins and peptides that were identified by mass spectrometry MS/MS. These studies reported the presence of molecules in the venom characterized as trypsin inhibitor-like protein and serine protease inhibitor protein [43]. 

The presence of serine proteases inhibitor transcripts in the venom glands of *Loxosceles* spiders was later identified in the transcriptome analysis of the venom-producing gland of *L. intermedia*, which showed the presence of 0.1% of toxin encoding transcripts identified as serine protease inhibitors [25]. Finally, a further biochemical characterization supporting the presence of serine protease inhibitors in the venom of *Loxosceles* spiders is currently being carried out by using a recombinant serine protease inhibitor from *L. intermedia* [100]. The reasons for the presence of serine protease inhibitors in the venom of *Loxosceles* spiders, as well as the physiological targets of these molecules are still unknown. It was proposed that these toxins, through their inhibitory activities on proteases, could protect the integrity of other venom components, and thus increase the useful life of venom toxins exposed to an external proteolytic environment, for instance when the venom is released to protect the spider against predators or to kill their prey [43]. 

An interesting fact that suggests the biological importance of serine protease inhibitors for *Loxosceles* spider venom is that toxins found in the venom of *L. laeta* are similar to serine protease inhibitors from different animals as *Mus musculus, Aedes aegypti, Branchiostoma lanceolatum, Gallus gallus* and *Boophilus microplus* [26]. This also is valid for serine protease inhibitors described in the *L. intermedia* venom, which are quite similar to inhibitors found in mammals such as *Mus musculus* and *Pan troglodytes*, or in the tick *Ambliomma americanum* [25]. These toxins fit into the families of molecules with low expression in the venoms of *Loxosceles* spiders [25] and perhaps that is why they have been little studied so far. However, the identification of transcripts coding for serpins present in the venom-producing glands, and the production of recombinant molecules as tools, will contribute and help elucidate the functions for these inhibitors, as well as contribute to structural analysis to understand the relation between structure and function of these toxins. Finally, but not less important is the possible uses of these molecules, since there are numerous examples of biotechnological applications of analogs of serpins in the control of blood clotting, anti-tumor activity and viral infection treatments [98,101-105]. 

### ICK Peptides or Knottins

Inhibitory Cystine Knot (ICK) peptides are single-chain molecules enriched in cysteine residues, which establish intramolecular disulfide bonds. The disulfide bonds are organized in a specific pattern in which two of them together with the peptide backbone form a ring that is crossed by the third disulfide bond. This disulfide bonds’ arrangement creates a pseudo-knot framework, which is why these peptides are also known as knottins [106-108]. A large number of studies have already shown that ICK peptides display insecticidal activity [109-111]. For this reason, the foremost function regarding ICK peptides in spider venoms concern the predation for feeding purposes, especially insects [112-114]. It is also due to this insecticidal activity that ICK peptides have been biotechnologically explored in order to develop alternative bioinsecticides to the harmful chemical compounds still used [113,115]. 

The ICK peptides from *Loxosceles* spiders were first studied by De Castro et al. [110] who fractioned the crude venom of *L. intermedia* and identified a fraction with insecticidal activity (Figure 3A). Further chromatographic steps sequentially performed allowed the purification of three peptides named LiTx1, LiTx2 and LiTx3 with insecticidal activity (induced flaccid paralysis) on the larvae of economic interest *Spodoptera frugiperda*. By using amino acid sequencing and molecular biology methodologies, De Castro et al. [110] obtained the coding sequence for these peptides, which revealed that these peptides are produced as prepropeptide precursors (signal peptide, propeptide and mature peptide). Later, the authors included a sequence related to a fourth isoform (LiTx4) on the GenBank, which has not had its insecticidal activity tested yet.

The transcriptome of the venom-producing glands of *L. intermedia* published by Gremski et al. [25] revealed that the majority of the sequences expressed regarding toxins was related to ICK peptides (55.9%). Sequences with high identity with the LiTx1-4 peptides represented 53.5%, and 2.4% of that transcripts showed significant similarity with a neurotoxic ICK peptide from the spider *Macrothele gigas* [116]. In addition to the annotation of the sequences mentioned, Gremski et al. [25] analyzed the venom protein content by SDS-PAGE, which suggested that ICK peptides are massively predominant in the venom of *L. intermedia*. 

Matsubara et al. [117] investigated an ICK peptide sharing 86% sequence identity with LiTx3, which was named U2-sicaritoxin-Li1b (U_2_-SCRTX-Li1b) in agreement with the rational nomenclature developed by King et al. [118]. The authors cloned the sequence and expressed the peptide in bacterial cells, resulting in the production and purification of the first recombinant ICK peptide from *Loxosceles*’ venoms. Using the recombinant U_2_-SCRTX-Li1b and hyperimmune sera raised against different *Loxosceles* spider venoms, the authors performed immunoassays that showed antigenic cross-reactivity, pointing out that ICK peptides constitute a family of toxins widespread throughout the genus [117]. An additional ELISA cross-reactivity analysis using polyclonal antibodies and the recombinant peptide U_2_-SCRTX-Li1b or whole venom performed by Buch et al. [91] suggested that ICK peptides are present in the venoms of *L. intermedia*, *L. laeta* and *L. gaucho*, reinforcing once more that these peptides belong to a conserved family of toxins in *Loxosceles* spiders. Interestingly, Buch et al. [91] also carried out western blotting analysis that did not show cross-reactivity between the recombinant peptide and polyclonal antibodies that recognize *L. laeta* and *L. gaucho* venoms or between the polyclonal antibodies that recognize the peptide U_2_-SCRTX-Li1b and *L. laeta* and *L. gaucho* whole venoms.

Meissner et al. [119] studied another ICK peptide from *L. intermedia*, whose sequence was identified in the venom gland transcriptome [25]. This peptide - U_2_-SCRTX-Lit2 - shares 52% identity with the toxin μ-hexatoxin-Mg2a (µ-HXTX-Mg2a) from *M. gigas*, in addition to the fact that both peptides contain 10 cysteine residues that establish the same disulfide bond connectivity pattern. A great deal of ICK peptides have been described as highly specific to insects, interacting with ion channels or membrane receptors in their nervous system and then resulting in paralysis and death. In order to investigate the target of U_2_-SCRTX-Lit2, Meissner et al. [119] performed molecular docking and dynamics analyses using a voltage-gated sodium channel from *Spodoptera litura* (tobacco cutworm), whose structure has already been determined. The choice of this target was also due to the fact that µ-HXTX-Mg2a was able to cause paralysis in *S. litura* and was associated with the inhibition of voltage-gated sodium channels (SlNaVSC) in synaptosome preparations obtained from cockroaches by binding to the site 3 of these channels [116]. Bioinformatics data showed that U_2_-SCRTX-Lit2 presents amino acid residues arranged in a pattern that suggests affinity to the site 3 of the SlNaVSC and revealed that the peptide may act as a steric blocker, hiding the gate access of these channels [119]. Sequence analyses comparisons have pointed out that the peptides LiTx3 and U_2_-SCRTX-Li1b may act on voltage-gated sodium channels as well [110,117].

Another study regarding ICK peptides from *L. intermedia* was carried out by Matsubara et al. [111]. In this study, the peptide U_2_-SCRTX-Li1b was recombinantly expressed in the periplasm of bacterial cells. This strategy was selected because the periplasm of *E. coli* provides the molecular machinery that assists in the correct formation of disulfide bridges, in contrast to the reducing environment of the cytoplasm that disadvantages the establishment of these structures [120]. After purification, recombinant U_2_-SCRTX-Li1b was able to cause long-lasting paralysis in sheep blowflies (*Lucilia cuprina*), which was irreversible even after 72 hours. Therefore, U_2_-SCRTX-Li1b constitutes the first recombinant ICK peptide from *Loxosceles* spiders to have its activity determined [111]. Furthermore, the authors carried out a screening of sequences encoding ICK peptides in other two *Loxosceles* species (*L. gaucho* and *L. laeta*) from the total RNA produced in the venom glands of the spiders. This screening of venom-gland transcripts resulted in the obtainment of sequences encoding orthologues of LiTx1-4 peptides, with identities ranging from 83% to 100% compared to the sequences encoding ICK peptides of *L. intermedia*. All sequences encoding ICK peptides found contain 10 cysteine residues in their mature sequence and exhibit the same predicted disulfide bond connectivity pattern [111].

In 2017, Trevisan-Silva et al [67] published a revealing proteomic analysis of the whole venom of *L. intermedia* by using a multi-protease, multi-dissociation, bottom-up-to-top-down approach. This study identified ICK peptides from *Loxosceles* venoms at a proteomic level for the first time, resulting in the identification of peptides with correspondence to LiTx in high abundance as depicted by the *L. intermedia* transcriptome [25].

ICK peptides have also been described as toxins that help spiders in defending against their predators. In addition, due to the anthropic action, many species of spiders have had their natural habitats destroyed and, consequently, they have been recurrently found in peridomiciliar environments, which facilitates accidents with humans [3,6]. Due to natural interactions with predators and episodic interactions with humans, many species of spiders have evolved ICK peptides with harmful properties to these organisms. *Loxosceles* bites result in a mild stinging that usually is painless in humans [3,6], which can suggest the existence of molecules with anesthetic or analgesic effects in the venom. Some ICK peptides in other spiders have already proved to display analgesic effects on animal models such as the peptide PcTx1 (µ-TRTX-Pc1a) from the tarantula *Psalmopoeus cambridgei*, which interacts with acid sensing ion channels and results in analgesic properties in rat models for acute pain when administered intrathecally or intracerebroventricularly [107,121]. Hence, given the great diversity of ICK peptide-coding sequences and the painless aspect of *Loxosceles* spider bites, the search for peptides with possible analgesic activities remains a promising idea. 

## Conclusion

Much learning has been gathered about astacins, serine proteases, knottins, TCTP, hyaluronidases, allergens and serpins (Table 1). However, we are still at an initial phase in understanding the full role of these proteins in the brown spider venom and how they can work together to affect the tissue of victims. Novel strategies must be undertaken to overcome the barrier of obtaining enough amount of these toxins to enable further investigation and comprehension of the pathophysiology of loxoscelism. Then, it will be possible to use this information to improve therapeutic strategies for treating affected patients. In addition, a deeper knowledge on functional and structural aspects of these poorly explored toxins will certainly reveal new possible applications in diverse areas.

**Table 1.** A summary of the studies involving each family of toxins present in the venom of *Loxosceles* spiders approached in this review.

Toxin	References	Major findings
Metalloproteases (Astacins)	Feitosa et al. [32], 1998	*L. intermedia* venom was able to degrade fibronectin and fibrinogen, but not laminin or types I and IV collagens. This activity was blocked by EDTA and 1,10-phenantroline. Zymogram analyzes of venom detected a 35 kDa enzyme with gelatinolytic activity and a fibronectinolytic and fibrinogenolytic band at 28 kDa.
	Da Silveira et al. [40], 2007	A 30 kDa metalloprotease was cloned and produced a recombinant protein in a prokaryotic expression system. It was named LALP1, from *Loxosceles* astacin-like protease, because it was structurally and functionally related to the astacin family of metalloproteases. LALP1 induced de-adhesion of endothelial cell cultures and degraded fibronectin and fibrinogen.
	Trevisan-Silva et al. [122], 2010	Two novel cDNAs encoding astacins were cloned from *L. intermedia* venom glands (LALP2 and LALP3). The venoms of *L. intermedia*, *L. laeta* and *L. gaucho* showed immunologically-related toxins with LALP1 and toxins with gelatinolytic activity with the same electrophoretic mobilities. The screening of mRNAs from *L. laeta* and *L. gaucho* venom glands revealed members of the astacin family (LALP4 and LALP5, respectively).
	Trevisan-Silva et al. [44], 2013	Based on the analysis of subproteomes of LALPs from *L. intermedia*, *L. laeta* and *L. gaucho*, authors showed that LALPs comprise a large family of toxins in *Loxosceles* venom, and that each venom has distinct proteolytic activities.
	Morgon et al. [46], 2017	LALP3 was expressed using a SUMO tag in *Escherichia coli* Shuffle T7 Express LysY cells. Immunoassays showed that LALP1 and LALP3 share linear epitopes and LALP3 shares conformational epitopes with native venom astacins. Molecular modeling of LALP3 revealed the zinc binding and Met-turn motifs forming the active site.
	Medina-Santos et al. [41], 2019	*L. laeta* venom gland transcripts were analyzed with a focus on LALPs and nine possible LALPs isoforms from Peruvian *L. laeta* venom were identified and validated by *in silico* and *in vitro* experiments.
Serine proteases	Veiga et al. [51], 2000	Serine protease activity was detected in the venom of *L. intermedia* after treatment with trypsin. These gelatinolytic molecules presented electrophoretic mobility of 85 and 95 kDa in a zymogram analysis.
Hyaluronidases	Da Silveira et al. [66], 2006	*L. intermedia* venom hyaluronidases were characterized as endo-β-N-acetyl-D-hexosaminidases that hydrolyze hyaluronic acid (HA) and chondroitin sulfate (CS). Lytic activities upon these GAGs were observed by zymogram analyzes at 41 and 43 kDa.
	Ferrer et al. [68], 2013	A recombinant hyaluronidase (Dietrich’s hyaluronidase) from *L. intermedia* venom was expressed and refolded. It was able to degrade HA and CS, cross-reacted with native venom toxins and increased the dermonecrotic effect of a *Loxosceles* PLD, confirming its activity as a spreading factor.
	De-Bona et al. [71], 2021	A novel hyaluronidase of *L. intermedia* venom was produced in a baculovirus-insect cell expression system as a fully active enzyme with post-translationally modifications (i.e., N-linked carbohydrates). LiHyal2, as it was named, potentialized dermonecrosis, edema and vascular permeability induced by a *Loxosceles* PLD.
Allergens	Justa et al. [19], 2020	A novel allergen toxin, named LALLT, was produced in a eukaryotic expression system and triggered paw edema and increased vascular permeability in mice. LALLT also induced erythema, edema and leukocyte infiltration in rabbit skin. The degranulation of mastocytes by LALLT was observed *in vivo* and *in vitro*. RNA screening indicated the presence of allergen toxins in the venom of other *Loxosceles* spiders.
Translationally controlled tumor protein (TCTP)	Sade et al. [87], 2012	A novel member of the TCTP family from *L. intermedia* venom gland was heterologously expressed and purified. LiTCTP caused edema and enhanced vascular permeability.
	Boia-Ferreira et al. [88], 2019	LiTCTP was characterized as an essential synergistic factor for the dermonecrotic toxin action, increasing the inflammatory response, capillary permeability, edema and contributing to the exacerbated inflammatory response.
ICK peptides	De Castro et al. [110], 2004	Native peptides with insecticidal activity (LiTx1, LiTx2 and LiTx3) were purified from *L. intermedia* venom by using chromatographic approaches.
	Matsubara et al. [117], 2013	U_2_-SCRTX-Li1b, an ICK peptide from *L. intermedia*, was heterologously expressed in bacteria - first ICK peptide from *Loxosceles* spiders to be produced in its recombinant form. Immunoblotting assays pointed out that ICK peptides constitute a family of toxins conserved in the genus.
	Meissner et al. [119], 2016	U_2_-SCRTX-Lit2, another ICK peptide of *L. intermedia*, was cloned. Molecular dynamics analysis revealed that U_2_-SCRTX-Lit2 possibly acts as a steric blocker, hiding the gate access of Na^+^-voltage-gated channels.
	Matsubara et al. [111], 2017	U_2_-SCRTX-Li1b was heterologously expressed in the periplasm of bacteria. U_2_-SCRTX-Li1b caused long-lasting paralysis in sheep blowflies, which was irreversible even after 72 h - first recombinant ICK peptide from *Loxosceles* spiders to have its activity determined. ICK peptides were identified from the RNA extracted from the venom-producing glands of *L. gaucho* and *L. laeta* - first study to identify ICK peptides in species other than *L. intermedia*.

## References

[B1] Saúde Ministério da (2020). Acidente por animais peçonhentos - Notificações registradas no Sistema de Informação de Agravos de Notificação - Brasil. SINAN.

[B2] Futrell JM (1992). Loxoscelism. Am J Med Sci.

[B3] da Silva PH, da Silveira RB, Helena Appel M, Mangili OC, Gremski W, Veiga SS (2004). Brown spiders and loxoscelism. Toxicon.

[B4] Cameron HD, American Arachnological Society (2005). An etymological dictionary of North American spider genus names. Spiders North Am An Identif Man.

[B5] Vetter RS, Rust MK (2008). Refugia Preferences by the Spiders Loxosceles reclusa and Loxosceles laeta (araneae: Sicariidae). J Med Entomol.

[B6] Gremski LH, Trevisan-Silva D, Ferrer VP, Matsubara FH, Meissner GO, Wille ACM (2014). Recent advances in the understanding of brown spider venoms: From the biology of spiders to the molecular mechanisms of toxins. Toxicon.

[B7] Platnick NI (2019). World Spider Catalog.

[B8] Málaque CMSA, Castro-Valencia JE, Cardoso JLC, França FO de S, Barbaro KC, Fan HW (2002). Clinical and epidemiological features of definitive and presumed loxoscelism in São Paulo, Brazil. Rev Inst Med Trop S Paulo.

[B9] Pace LB, Vetter RS (2009). Brown recluse spider (Loxosceles reclusa) envenomation in small animals. J Vet Emerg Crit Care (San Antonio).

[B10] Isbister GK, Fan HW (2011). Spider bite. Lancet.

[B11] Smith CW, Micks DW (1970). The role of polymorphonuclear leukocytes in the lesion caused by the venom of the brown spider, Loxosceles reclusa. Lab Invest.

[B12] Yiannias JA, Winkelmann RK (1992). Persistent painful plaque due to a brown recluse spider bite. Cutis.

[B13] Elston DM, Eggers JS, Schmidt WE, Storrow AB, Doe RH, McGlasson D (2000). Histological findings after brown recluse spider envenomation. Am J Dermatopathol.

[B14] Sunderkötter C, Seeliger S, Schönlau F, Roth J, Hallmann R, Luger TA (2001). Different pathways leading to cutaneous leukocytoclastic vasculitis in mice. Exp Dermatol.

[B15] Ospedal KZ, Appel MH, F JF, Mangili OC, Veiga SS, Gremski W (2002). Histopathological findings in rabbits after experimental acute exposure to the Loxosceles intermedia (brown spider) venom. Int J Exp Pathol.

[B16] Patel KD, Modur V, Zimmerman GA, Prescott SM, McIntyre TM (1994). The necrotic venom of the brown recluse spider induces dysregulated endothelial cell-dependent neutrophil activation. Differential induction of GM-CSF, IL-8, and E-selectin expression. J Clin Invest.

[B17] Gremski LH, Da Justa HC, Da Silva TP, Polli NLC, Antunes BC, Minozzo JC (2020). Forty years of the description of brown spider venom phospholipases-D. Toxins (Basel).

[B18] Hogan CJ, Barbaro KC, Winkel K (2004). Loxoscelism: old obstacles, new directions. Ann Emerg Med.

[B19] Justa HC, Matsubara FH, de-Bona E, Schemczssen-Graeff Z, Polli NLC, de Mari TL (2020). LALLT (Loxosceles Allergen-Like Toxin) from the venom of Loxosceles intermedia: Recombinant expression in insect cells and characterization as a molecule with allergenic properties. Int J Biol Macromol.

[B20] Vetter RS (2011). Seasonality of brown recluse spiders, Loxosceles reclusa, submitted by the general public: Implications for physicians regarding loxoscelism diagnoses. Toxicon.

[B21] Lucato RV, Abdulkader RCRM, Barbaro KC, MendesGlória GE, Castro I, Baptista MASF (2011). Loxosceles gaucho venom-induced acute kidney injury - in vivo and in vitro studies. PLoS Negl Trop Dis.

[B22] Sams HH, Dunnick CA, Smith ML, King LEJr (2001). Necrotic arachnidism. J Am Acad Dermatol.

[B23] Binford GJ, Wells MA (2003). The phylogenetic distribution of sphingomyelinase D activity in venoms of Haplogyne spiders. Comp Biochem Physiol - B Biochem Mol Biol.

[B24] Dos Santos VLP, Franco CRC, Viggiano RLL, Da Silveira RB, Cantão MP, Mangili OC (2000). Structural and ultrastructural description of the venom gland of Loxosceles intermedia (brown spider). Toxicon.

[B25] Gremski LH, Da Silveira RB, Chaim OM, Probst CMA, Ferrer VP, Nowatzki J (2010). A novel expression profile of the Loxosceles intermedia spider venomous gland revealed by transcriptome analysis. Mol Biosyst.

[B26] Fernandes-Pedrosa MF, Junqueira-de-Azevedo ILM, Gonçalves-de-Andrade RM, Kobashi LS, Almeida DD, Ho PL (2008). Transcriptome analysis of Loxosceles laeta (Araneae, Sicariidae) spider venomous gland using expressed sequence tags. BMC Genomics.

[B27] Dantas AE, Carmo AO, Horta CCR, Leal HG, Oliveira-Mendes BBR, Martins APV (2016). Description of Loxtox protein family and identification of a new group of Phospholipases D from Loxosceles similis venom gland. Toxicon.

[B28] Chaves-Moreira D, Senff-Ribeiro A, Wille ACM, Gremski LH, Chaim OM, Veiga SS (2017). Highlights in the knowledge of brown spider toxins. J Venom Anim Toxins incl Trop Dis.

[B29] Fingermann M, de Roodt AR, Cascone O, Miranda MV (2020). Biotechnological potential of Phospholipase D for Loxosceles antivenom development. Toxicon X.

[B30] Eskafi FM, Norment BR (1976). Physiological action of Loxosceles reclusa (G&M) venom on insect larvae. Toxicon.

[B31] Jong YS, Norment BR, Heitz JR (1979). Separation and characterization of venom components in Loxosceles reclusa-II. Protease enzyme activity. Toxicon.

[B32] Feitosa L, Gremski W, Veiga SS, Elias MCQB, Graner E, Mangili OC (1998). Detection and characterization of metalloproteinases with gelatinolytic, fibronectinolytic and fibrinogenolytic activities in Brown spider (Loxosceles intermedia) venom. Toxicon.

[B33] Veiga SS, Zanetti VC, Braz A, Mangili OC, Gremski W (2001). Extracellular matrix molecules as targets for brown spider venom toxins. Braz J Med Biol Res.

[B34] Veiga SS, Zanetti VC, Franco CRC, Trindade ES, Porcionatto MA, Mangili OC (2001). In vivo and in vitro cytotoxicity of brown spider venom for blood vessel endothelial cells. Thromb Res.

[B35] Zanetti VC, Da Silveira RB, Dreyfuss JL, Haoach J, Mangili OC, Veiga SS (2002). Morphological and biochemical evidence of blood vessel damage and fibrinogenolysis triggered by brown spider venom. Blood Coagul Fibrinolysis.

[B36] Barretto OCO, Satake M, Nonoyama K, Cardoso JLC (2003). The calcium-dependent protease of Loxosceles gaucho venom acts preferentially upon red cell band 3 transmembrane protein. Braz J Med Biol Res.

[B37] Young AR, Pincus SJ (2001). Comparison of enzymatic activity from three species of necrotising arachnids in Australia: Loxosceles rufescens, Badumna insignis and Lampona cylindrata. Toxicon.

[B38] Barbaro KC, Knysak I, Martins R, Hogan C, Winkel K (2005). Enzymatic characterization, antigenic cross-reactivity and neutralization of dermonecrotic activity of five Loxosceles spider venoms of medical importance in the Americas. Toxicon.

[B39] Da Silveira RB, Dos Santos JF, Mangili OC, Veiga SS, Gremski W, Nader HB (2002). Identification of proteases in the extract of venom glands from brown spiders. Toxicon.

[B40] da Silveira RB, Wille ACM, Chaim OM, Appel MH, Silva DT, Franco CRC (2007). Identification, cloning, expression and functional characterization of an astacin-like metalloprotease toxin from Loxosceles intermedia (brown spider) venom. Biochem J.

[B41] Calabria PAL, Shimokava-Falcao LHAL, Colombini M, Moura-da-Silva AM, Barbaro KC, Faquim-Mauro EL (2019). Design and production of a recombinant hybrid toxin to raise protective antibodies against Loxosceles spider venom. Toxins (Basel).

[B42] Medina-Santos R, Guerra-Duarte C, de Almeida Lima S, Costal-Oliveira F, Alves de Aquino P, Oliveira do Carmo A (2019). Diversity of astacin-like metalloproteases identified by transcriptomic analysis in Peruvian Loxosceles laeta spider venom and in vitro activity characterization. Biochimie.

[B43] dos Santos LD, Dias NB, Roberto J, Pinto AS, Palma MS (2009). Brown recluse spider venom: proteomic analysis and proposal of a putative mechanism of action. Protein Pept Lett.

[B44] Trevisan-Silva D, Gremski LH, Chaim OM, Senff-Ribeiro A, Veiga SS (2013). Loxosceles Astacin-Like Proteases (LALPs). Handb Proteolytic Enzym.

[B45] Trevisan-Silva D, Bednaski AV, Gremski LH, Chaim OM, Veiga SS, Senff-Ribeiro A (2013). Differential metalloprotease content and activity of three Loxosceles spider venoms revealed using two-dimensional electrophoresis approaches. Toxicon.

[B46] Morgon AM, Belisario-Ferrari MR, Trevisan-Silva D, Meissner GO, Vuitika L, Marin B (2016). Expression and immunological cross-reactivity of LALP3, a novel astacin-like metalloprotease from brown spider (Loxosceles intermedia) venom. Biochimie.

[B47] Costa TGF, Costal-Oliveira F, de Assis TCS, Lima SA, Martins CA, Finco AB (2020). Engineered antigen containing epitopes from Loxosceles spp. spider toxins induces a monoclonal antibody (Lox-mAb3) against astacin-like metalloproteases. Int J Biol Macromol.

[B48] Macías-Hernández N, Athey K, Tonzo V, Wangensteen OS, Arnedo M, Harwood JD (2018). Molecular gut content analysis of different spider body parts. PLoS One.

[B49] Veiga SS, Feitosa L, dos Santos VLP, de Souza GA, Ribeiro AS, Mangili OC (2000). Effect of brown spider venom on basement membrane structures. Histochem J.

[B50] Lima S de A, Guerra-Duarte C, Costal-Oliveira F, Mendes TM, Luís LF, Oliveira D (2018). Recombinant protein containing B-cell epitopes of different Loxosceles spider toxins generates neutralizing antibodies in immunized rabbits. Front Immunol.

[B51] Veiga SS, Da Silveira RB, Dreyfuss JL, Haoach J, Pereira AM, Mangili OC (2000). Identification of high molecular weight serine-proteases in Loxosceles intermedia (brown spider) venom. Toxicon.

[B52] Kaiser E (1953). The enzymatic activity of spider venom. Mem Inst Butantan.

[B53] Kuhn-Nentwig L, Schaller J, Nentwig W (1994). Purification of toxic peptides and the amino acid sequence of CSTX-1 from the multicomponent venom of Cupiennius salei (Araneae:Ctenidae). Toxicon.

[B54] Rash LD, Hodgson WC (2002). Pharmacology and biochemistry of spider venoms. Toxicon.

[B55] Nagaraju S, Devaraja S, Kemparaju K (2007). Purification and properties of hyaluronidase from Hippasa partita (funnel web spider) venom gland extract. Toxicon.

[B56] Rocha-e-Silva TAA, Sutti R, Hyslop S (2009). Milking and partial characterization of venom from the Brazilian spider Vitalius dubius (Theraphosidae). Toxicon.

[B57] Fox JW (2013). A brief review of the scientific history of several lesser-known snake venom proteins: L-amino acid oxidases, hyaluronidases and phosphodiesterases. Toxicon.

[B58] Gouveia AIDCB, Da Silveira RB, Nader HB, Dietrich CP, Gremski W, Veiga SS (2005). Identification and partial characterisation of hyaluronidases in Lonomia obliqua venom. Toxicon.

[B59] Kemeny DM, Dalton N, Lawrence AJ, Pearce FL, Vernon CA (1984). The purification and characterisation of hyaluronidase from the venom of the honey bee, Apis mellifera. Eur J Biochem.

[B60] Frangieh J, Salma Y, Haddad K, Mattei C, Legros C, Fajloun Z (2019). First characterization of the venom from Apis mellifera syriaca, a honeybee from the middle east region. Toxins (Basel).

[B61] Hall JE (1970). A study of protein and peptide components of venoms of *Loxosceles reclusa* Gertsch and Mulaik and Dugesiella hentzi (Girard).

[B62] Wright RP, Elgert KD, Campbell BJ, Barrett JT (1973). Hyaluronidase and esterase activities of the venom of the poisonous brown recluse spider. Arch Biochem Biophys.

[B63] Elgert KD, Ross MA, Campbell BJ, Barrett JT (1974). Immunological studies of brown recluse spider venom. Infect Immun.

[B64] Geren CR, Chan TK, Howell DE, Odell GV (1976). Isolation and characterization of toxins from brown recluse spider venom (Loxosceles reclusa). Arch Biochem Biophys.

[B65] Schenone H, Suarez G (1978). Venoms of Scytodidae. Genus *Loxosceles*. Arthropod Venoms.

[B66] da Silveira RB, Chaim OM, Mangili OC, Gremski W, Dietrich CP, Nader HB (2007). Hyaluronidases in Loxosceles intermedia (Brown spider) venom are endo-β-N-acetyl-d-hexosaminidases hydrolases. Toxicon.

[B67] Trevisan-Silva D, Bednaski AV, Fischer JSG, Veiga SS, Bandeira N, Guthals A (2017). A multi-protease, multi-dissociation, bottom-up-to-top-down proteomic view of the Loxosceles intermedia venom. Sci Data.

[B68] Ferrer VP, de Mari TL, Gremski LH, Trevisan Silva D, da Silveira RB, Gremski W (2013). A Novel Hyaluronidase from Brown Spider (Loxosceles intermedia) Venom (Dietrich’s Hyaluronidase): From Cloning to Functional Characterization. PLoS Negl Trop Dis.

[B69] Clement H, Olvera A, Rodríguez M, Zamudio F, Palomares LA, Possani LD (2012). Identification, cDNA cloning and heterologous expression of a hyaluronidase from the tarantula Brachypelma vagans venom. Toxicon.

[B70] Biner O, Trachsel C, Moser A, Kopp L, Langenegger N, Kämpfer U (2015). Isolation, N-glycosylations and function of a hyaluronidase-like enzyme from the venom of the spider Cupiennius salei. PLoS One.

[B71] De-Bona E, Chaves-Moreira D, Batista TBD, Justa HC da, Rossi GR, Antunes BC (2021). Production of a novel recombinant brown spider hyaluronidase in baculovirus-infected insect cells. Enzyme Microb Technol.

[B72] Locke KW, Maneval DC, LaBarre MJ (2019). ENHANZE® drug delivery technology: a novel approach to subcutaneous administration using recombinant human hyaluronidase PH20. Drug Deliv.

[B73] Van Paassen P, Pittrow D, Scheidegger C, Klotsche J, Ellerbroek PM (2020). Use of recombinant human hyaluronidase-facilitated subcutaneous immunoglobulin in elderly patients. Immunotherapy.

[B74] Juhász MLW, Levin MK, Marmur ES (2017). The Kinetics of Reversible Hyaluronic Acid Filler Injection Treated with Hyaluronidase. Dermatol Surg.

[B75] Girish KS, Kemparaju K (2007). The magic glue hyaluronan and its eraser hyaluronidase: A biological overview. Life Sci.

[B76] Bordon KCF, Wiezel GA, Amorim FG, Arantes EC (2015). Arthropod venom Hyaluronidases: Biochemical properties and potential applications in medicine and biotechnology. J Venom Anim Toxins incl Trop Dis.

[B77] Bernton HS, Brown H (1964). Insect allergy-Preliminary studies of the cockroach. J Allergy.

[B78] Hoffman DR (2010). Ant venoms. Curr Opin Allergy Clin Immunol.

[B79] Perez-Riverol A, Justo-Jacomini DL, de Lima Zollner R, Brochetto-Braga MR (2015). Facing hymenoptera venom allergy: from natural to recombinant allergens. Toxins (Basel).

[B80] Pomés A, Chapman MD, Wünschmann S (2016). Indoor Allergens and Allergic Respiratory Disease. Curr Allergy Asthma Rep.

[B81] Cui Y, Yu L, Teng F, Wang N, Zhou Y, Zhang C (2018). Expression of recombinant allergen, der f 1, der f 2 and der f 4 using baculovirus-insect cell systems. Arch Med Sci.

[B82] Makris M, Spanoudaki N, Giannoula F, Chliva C, Antoniadou A, Kalogeromitros D (2009). Acute generalized exanthematous pustulosis (AGEP) triggered by a spider bite. Allergol Int.

[B83] Lane L, McCoppin HH, Dyer J (2011). Acute generalized exanthematous pustulosis and coombs-positive hemolytic anemia in a child following Loxosceles reclusa envenomation. Pediatr Dermatol.

[B84] Miteva M, Elsner P, Ziemer M (2009). A histopathologic study of arthropod bite reactions in 20 patients highlights relevant adnexal involvement. J Cutan Pathol.

[B85] MacDonald SM, Rafnar T, Langdon J, Lichtenstein LM (1995). Molecular identification of an IgE-dependent histamine-releasing factor. Science.

[B86] Bartley K, Nisbet AJ, Offer JE, Sparks NHC, Wright HW, Huntley JF (2009). Histamine Release Factor from Dermanyssus gallinae (De Geer): Characterization and in vitro assessment as a protective antigen. Int J Parasitol.

[B87] Sade YB, Bóia-Ferreira M, Gremski LH, Da Silveira RB, Gremski W, Senff-Ribeiro A (2012). Molecular cloning, heterologous expression and functional characterization of a novel translationally-controlled tumor protein (TCTP) family member from Loxosceles intermedia (brown spider) venom. Int J Biochem Cell Biol.

[B88] Boia-Ferreira M, Moreno KG, Basílio ABC, da Silva LP, Vuitika L, Soley B (2019). TCTP from Loxosceles Intermedia (Brown Spider) Venom Contributes to the Allergic and Inflammatory Response of Cutaneous Loxoscelism. Cells.

[B89] Rattmann YD, Pereira CR, Cury Y, Gremski W, Marques MCA, da Silva-Santos JE (2008). Vascular permeability and vasodilation induced by the Loxosceles intermedia venom in rats: Involvement of mast cell degranulation, histamine and 5-HT receptors. Toxicon.

[B90] Paludo KS, Biscaia SMP, Chaim OM, Otuki MF, Naliwaiko K, Dombrowski PA (2009). Inflammatory events induced by brown spider venom and its recombinant dermonecrotic toxin: A pharmacological investigation. Comp Biochem Physiol - C Toxicol Pharmacol.

[B91] Buch DR, Souza FN, Meissner GO, Morgon AM, Gremski LH, Ferrer VP (2015). Brown spider (Loxosceles genus) venom toxins: Evaluation of biological conservation by immune cross-reactivity. Toxicon.

[B92] Kimura T, Ono S, Kubo T (2012). Molecular cloning and sequence analysis of the cDNAS encoding toxin-like peptides from the venom glands of tarantula Grammostola rosea. Int J Pept.

[B93] Zobel-Thropp PA, Correa SM, Garb JE, Binford GJ (2014). Spit and venom from scytodes spiders: A diverse and distinct cocktail. J Proteome Res.

[B94] Zhang Y, Chen J, Tang X, Wang F, Jiang L, Xiong X (2010). Transcriptome analysis of the venom glands of the Chinese wolf spider Lycosa singoriensis. Zoology (Jena).

[B95] Jiang L, Zhang D, Zhang Y, Peng L, Chen J, Liang S (2010). Venomics of the spider Ornithoctonus huwena based on transcriptomic versus proteomic analysis. Comp Biochem Physiol - Part D Genomics Proteomics.

[B96] Mulenga A, Azad AF (2005). The molecular and biological analysis of ixodid ticks histamine release factors. Exp Appl Acarol.

[B97] Senff-Ribeiro A, Telerman A, Amson R (2017). Translattionaly controlled tumor protein (TCTP/HRF) in Animal Venoms. TCTP/tpt1 - Remodel Signal from Stem Cell to Dis Results Probl Cell Differ.

[B98] Chaves-Moreira D, Matsubara FH, Schemczssen-Graeff Z, De Bona E, Heidemann VR, Guerra-Duarte C (2019). Brown Spider (Loxosceles) Venom Toxins as Potential Biotools for the Development of Novel Therapeutics. Toxins (Basel).

[B99] Kniker WT, Morgan PN, Flanigan WJ, Reagan PW, Dillaha CJ (1969). An inhibitor of complement in the venom of the brown recluse spider, Loxosceles reclusa. Proc Soc Exp Biol Med.

[B100] Graeff ZS, Nowatzki J, de Bona E, Da Justa HC, Gremski LH, Veiga SS (2019). Molecular cloning and characterization of a serpin from Loxosceles intermedia venom gland. Toxicon.

[B101] Carneiro-Lobo TC, Konig S, Machado DE, Nasciutti LE, Forni MF, Francischetti IMB (2009). Ixolaris, a tissue factor inhibitor, blocks primary tumor growth and angiogenesis in a glioblastoma model. J Thromb Haemost.

[B102] Liu Z, Deng M, Xiang J, Ma H, Hu W, Zhao Y (2012). A Novel Spider Peptide Toxin Suppresses Tumor Growth Through Dual Signaling Pathways. Curr Mol Med.

[B103] Wan H, Lee KS, Kim BY, Zou FM, Yoon HJ, Je YH (2013). A Spider-Derived Kunitz-Type Serine Protease Inhibitor That Acts as a Plasmin Inhibitor and an Elastase Inhibitor. PLoS One.

[B104] Zhou Y, Vedantham P, Lu K, Agudelo J, Carrion R, Nunneley JW (2015). Protease inhibitors targeting coronavirus and filovirus entry. Antiviral Res.

[B105] Hoffmann M, Kleine-Weber H, Schroeder S, Krüger N, Herrler T, Erichsen S (2020). SARS-CoV-2 Cell Entry Depends on ACE2 and TMPRSS2 and Is Blocked by a Clinically Proven Protease Inhibitor. Cell.

[B106] Norton RS, Pallaghy PK (1998). The cystine knot structure of ion channel toxins and related polypeptides. Toxicon.

[B107] Saez NJ, Senff S, Jensen JE, Er SY, Herzig V, Rash LD (2010). Spider-venom peptides as therapeutics. Toxins (Basel).

[B108] Dongol Y, Cardoso FC, Lewis RJ (2019). Spider knottin pharmacology at voltage-gated sodium channels and their potential to modulate pain Pathways. Toxins (Basel).

[B109] Herzig V, Araujo AD, Greenwood KP, Chin YKY, Windley MJ, Chong Y (2018). Evaluation of Chemical Strategies for Improving the Stability and Oral Toxicity of Insecticidal Peptides. Biomedicines.

[B110] De Castro CS, Silvestre FG, Araújo SC, Yazbeck GDM, Mangili OC, Cruz I (2004). Identification and molecular cloning of insecticidal toxins from the venom of the brown spider Loxosceles intermedia. Toxicon.

[B111] Matsubara FH, Meissner GO, Herzig V, Justa HC, Dias BCL, Trevisan-Silva D (2017). Insecticidal activity of a recombinant knottin peptide from Loxosceles intermedia venom and recognition of these peptides as a conserved family in the genus. Insect Mol Biol.

[B112] King GF, Hardy MC (2013). Spider-venom peptides: Structure, pharmacology, and potential for control of insect pests. Annu Rev Entomol.

[B113] King GF (2019). Tying pest insects in knots: the deployment of spider-venom-derived knottins as bioinsecticides. Pest Manag Sci.

[B114] Chaim OM, Trevisan-Silva D, Chaves-Moreira D, Wille ACM, Ferrer VP, Matsubara FH (2011). Brown spider (Loxosceles genus) venom toxins: Tools for biological purposes. Toxins (Basel).

[B115] Windley MJ, Herzig V, Dziemborowicz SA, Hardy MC, King GF, Nicholson GM (2012). Spider-venom peptides as bioinsecticides. Toxins (Basel).

[B116] Corzo G, Gilles N, Satake H, Villegas E, Dai L, Nakajima T (2003). Distinct primary structures of the major peptide toxins from the venom of the spider Macrothele gigas that bind to sites 3 and 4 in the sodium channel. FEBS Lett.

[B117] Matsubara FH, Gremski LH, Meissner GO, Constantino Lopes ES, Gremski W, Senff-Ribeiro A (2013). A novel ICK peptide from the Loxosceles intermedia (brown spider) venom gland: Cloning, heterologous expression and immunological cross-reactivity approaches. Toxicon.

[B118] King GF, Gentz MC, Escoubas P, Nicholson GM (2008). A rational nomenclature for naming peptide toxins from spiders and other venomous animals. Toxicon.

[B119] Meissner GO, de Resende Lara PT, Scott LPB, Braz ASK, Chaves-Moreira D, Matsubara FH (2016). Molecular cloning and in silico characterization of knottin peptide, U2-SCRTX-Lit2, from brown spider (Loxosceles intermedia) venom glands. J Mol Model.

[B120] Klint JK, Senff S, Saez NJ, Seshadri R, Lau HY, Bende NS (2013). Production of Recombinant Disulfide-Rich Venom Peptides for Structural and Functional Analysis via Expression in the Periplasm of E. coli. PLoS One.

[B121] Mazzuca M, Heurteaux C, Alloui A, Diochot S, Baron A, Voilley N (2007). A tarantula peptide against pain via ASIC1a channels and opioid mechanisms. Nat Neurosci.

[B122] Trevisan-Silva D, Gremski LH, Chaim OM, da Silveira RB, Meissner GO, Mangili OC (2010). Astacin-like metalloproteases are a gene family of toxins present in the venom of different species of the brown spider (genus Loxosceles). Biochimie.

[B123] Vuitika L, Chaves-Moreira D, Caruso I, Lima MA, Matsubara FH, Murakami MT (2016). Active site mapping of Loxosceles phospholipases D: Biochemical and biological features. Biochim Biophys Acta.

